# Supplementary material for: Determinants of care-seeking practice for neonatal illnesses in rural Bangladesh: A community-based cross-sectional study
Source: PLoS One. 2020 Oct 14;15(10):e0240316. doi: 10.1371/journal.pone.0240316 (PMC7556439; doi:10.1371/journal.pone.0240316)
Supplement: Attachment — Submitted filename: Comment PLOS.docx [file pone.0240316.s002.docx]

**Comments to the authors**

This study is interesting. However, this need to focus some areas:

1. In Background of Abstract, how poor utilization of skilled care services in neonatal illnesses is crucial to 18 reduce neonatal morbidity and mortality?

2. In second para of Introduction, “Available literature has reported that effective utilization of skilled health care services for 67 newborns remains low” where the research conducted?

3. In the same para “although the proportion of home-based delivery is reduced significantly” what does it mean kind of poor structure of the sentence.

4. Poor level of English and inconsistencies appeared in introduction section.

5. What type of individual and social factors are responsible for the utilization of a skilled health care provider?

6. This study did not provide any previous evidence however some literatures addressed this issue in Bangladesh and other countries.

7. Study gap is not clear including research question and theoretical framework.

8. In methods, sample size selection is not clear, how the authors selected sample, like, any formula, exclusion and inclusion criteria etc.

9. In discussion, line 245, the influential role of cultural beliefs and social stigma… what types of cultural beliefs and social stigma…

10. in line 246, “severe consequences” what are they

11. what are the new policies add to this study.

12. What is the difference between this study and “Chowdhury SK, Billah SM, Arifeen SE, Hoque DME (2018) Care-seeking practices for sick neonates: Findings from cross-sectional survey in 14 rural sub-districts of Bangladesh. PLoS ONE 13(9): e0204902”…

13. Overall cross check the table and text values and need comprehensive English language correction
