# Supplementary material for: Determinants of care-seeking practice for neonatal illnesses in rural Bangladesh: A community-based cross-sectional study
Source: PLoS One. 2020 Oct 14;15(10):e0240316. doi: 10.1371/journal.pone.0240316 (PMC7556439; doi:10.1371/journal.pone.0240316)
Supplement: Attachment — Submitted filename: PLOS One.docx [file pone.0240316.s003.docx]

**PLOS One: Title: Determinants of Care-seeking Practice for Neonatal Illnesses in Rural Bangladesh: A Community-based Cross-sectional Study**

Thank you for the opportunity to review the manuscript titled " Determinants of Care-seeking Practice for Neonatal Illnesses in Rural Bangladesh: A Community-based Cross-sectional Study". This manuscript examines the factors associated with seeking care from skilled healthcare service providers for reported neonatal illnesses in rural Matlab, Bangladesh

Overall, this manuscript can contribute to readers understanding on Care-seeking Practice for Neonatal Illnesses and research gap in the literature. However, this manuscript lacks of important components in methodology, analysis' description and discussion.  My review of the manuscript and recommendations to the authors are enclosed.

**Abstract**

Line 19, 27**: Comment**: Objectives and findings of the study are matched with a study conducted in rural Bangladesh in 2001 by Ahmed S et al.

Reference:Ahmed S, Sobhan F, Islam A. Neonatal morbidity and care‐seeking behavior in rural Bangladesh. Journal of tropical pediatrics. 2001;47(2):98-105

**Line 23:** Comment:Please mention the name of the method that helps to find the adjusted odds ratio.

**Line 23;** the sentence should be rephrased

**Introduction;** The introduction part has space to improve significantly with more relevant and recent references. The is also gap in consistence of the writing flow.

**Materials and methods**

Line:117: **Comment:** Please discuss the technique of selecting 2483 women randomly from 6,741 women. Without any explanation of a specific reason, there will be the possibility of selection bias.

Line 143: **Comment:** Add PNC visit as a covariate because it has an impact on mother knowledge about neonates illness. See the reference

Reference : Bulto GA, Fekene DB, Moti BE, Demissie GA, Daka KB. Knowledge of neonatal danger signs, care seeking practice and associated factors among postpartum mothers at public health facilities in ambo town, Central Ethiopia. BMC research notes. 2019;12(1):549.

**Line 159:** Comment: Please mention which model selection technique was followed to select determinants. The method that was used has the problem of overestimation. It didn’t say the way of optimizing the overestimation problem.

**Line 160: Comment:** Please mention the covariates in the method section or table that was used in the final model to get adjusted odds ratios

**Result:**

**Line 172, 173 Comment:** Mean age and median year of schooling were not found in table 1. Please check is it the number of school attendance or year of school attendance?

Line 180: Comment: Repetition

**Discussion:**

Discussion is too long, please try to reduce the texts. Please use recent reference from similar settings. Need to improve the language as well.

Line 214, 215, 216

Comment: Repetition of result

Line 267

Comment: Not only ANC but also PNC helps to strengthen mother knowledge about danger sign of neonate. See the below reference

Referance : Bulto GA, Fekene DB, Moti BE, Demissie GA, Daka KB. Knowledge of neonatal danger signs, care seeking practice and associated factors among postpartum mothers at public health facilities in ambo town, Central Ethiopia. BMC research notes. 2019;12(1):549.

Better to include **strength** and **Limitation** of the study

Conclusion: Conclusion should be based on study findings.
